# An Uncommon Twist: Isolated Fallopian Tube Torsion in an Adolescent

**DOI:** 10.1155/2013/509424

**Published:** 2013-08-19

**Authors:** Sundeep Kisku, Reju Joseph Thomas

**Affiliations:** Department of Paediatric Surgery, Christian Medical College, Vellore, Tamil Nadu 632004, India

## Abstract

We report a 13-year-old girl with bilateral paratubal cysts and left isolated fallopian tube torsion (IFTT). Paratubal cysts are uncommon in children, and IFTT is a rare complication. Awareness of this entity and prompt surgical intervention could potentially salvage the fallopian tube preserving fertility.

## 1. Introduction

Paratubal cysts occur in about 4% of adolescents [1] and can rarely undergo torsion along with the adnexal structures. Isolated fallopian tube torsion (IFTT) occurs when the fallopian tube torses without ovarian torsion. The incidence is 1 in 1 500 000 women [2]. Delay in diagnosis and treatment occur due to lack of definitive clinical features. Awareness of this entity, early diagnosis, and prompt treatment can salvage the torted fallopian tube and preserve fertility.

## 2. Case History

A 13-year-old postpubertal girl presented with vomiting, left flank, and lower abdominal pain for 5 days. She reported a similar, albeit milder episode 3 months ago. She was being treated by the emergency department for suspected urinary tract infection. On examination, she was afebrile and hemodynamically stable. There was tenderness in the left iliac fossa and differential tenderness on rectal examination, in the Pouch of Douglas.

Her hemoglobin was 11.0 gm% and total leukocyte count 10,600 cells/cc (66% polymorphs, 34% lymphocytes). Urine microscopy revealed RBC 15–20 cells/high power field (HPF) and WBC 6–8 cells/HPF. Abdominal sonography revealed a right ovarian cyst 6.4 × 3.8 × 5.4 cm.

**Figure 1:**
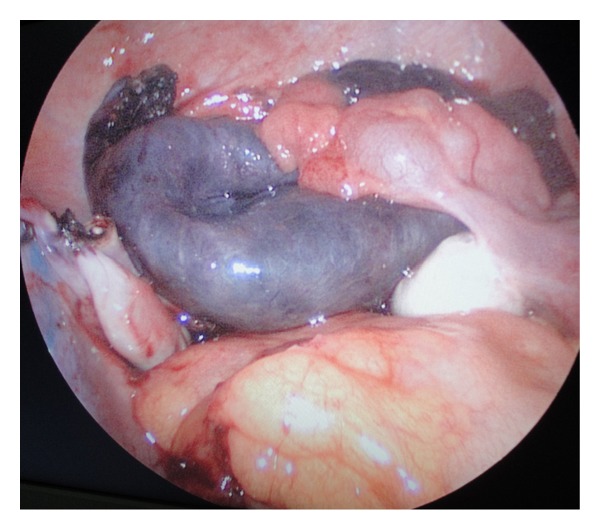
Normal ovaries with gangrene of the distal third of the left fallopian tube and paratubal cyst (postdetorsion and excision view). The right fallopian tube is seen with a few small paratubal cysts.

**Figure 2:**
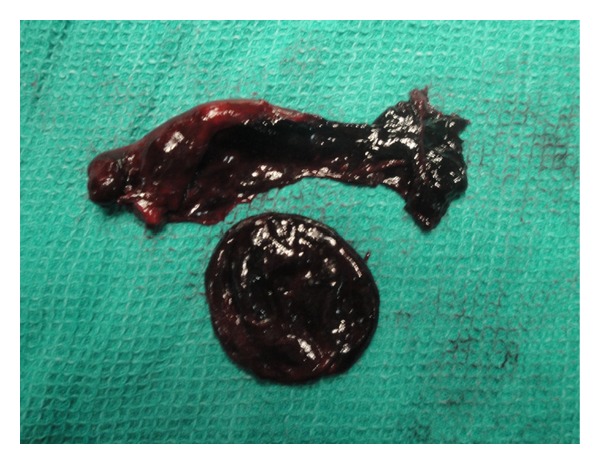
Left fallopian tube with paratubal cyst.

With a provisional diagnosis of torsion of the ovary, emergency laparoscopy was performed. The distal third of the left fallopian tube along with a 5 × 5 cm paratubal cyst (Figures 1 and 2) had undergone gangrenous torsion (2.5 turns). Both ovaries were normal. The right fallopian tube had several paratubal cysts in its distal third. The torted left cyst contained 40 mL of hemorrhagic fluid which was aspirated. The nonviable left fallopian tube and cyst were detorted and excised. The right paratubal cysts (three) were punctured, and clear fluid was drained. Histopathology revealed the left fallopian tube and cyst to be extensively congested, hemorrhagic, and infarcted.

The postoperative period was uneventful, and she was discharged two days later. The parents were counseled of possible infertility in the future due to fallopian tubal stenosis secondary to the paratubal cysts.

## 3. Discussion

IFTT occurs mostly in 12–15 year olds and is rare before menarche or during menopause. IFTT may occur due to intrinsic and extrinsic causes [3]. Intrinsic causes include congenital anomalies (long or spiral tube) acquired pathology (hydrosalpinx, cysts, pelvic inflammatory disease) and autonomic dysfunction with abnormal peristalsis. Extrinsic causes include local factors and mechanical factors, jarring movements of the body (Sellheim theory).

Paratubal cysts arise from Mullerian or Wolffian structures and are common in adult females (92% of hysterectomies). These are hormone sensitive and are generally asymptomatic [1]. However, these are uncommon in children. Hemorrhage, rupture, and infertility are rare complications and occur in large cysts. IFTT has been reported in with unilateral [4] and bilateral [5] paratubal cysts in older children. Small cysts (<3 cm) may be punctured and larger ones excised. Malignant neoplasms arising from paratubal cysts are very rare [6]. Regular followup with abdominal sonogram is advisable.

The most common symptom is ipsilateral lower abdominal or flank pain. Physical findings include abdominal tenderness with or without peritoneal signs and adnexal tenderness on rectal examination. A mass may not be palpable. Laboratory values are usually nonspecific.

The sonographic findings include normal appearing ovaries, free fluid, a dilated tube with thickened, echogenic walls, and internal debris representing a torsed tube [7]. A whirlpool sign specific of IFTT has been described [8]. Paratubal cysts are difficult to diagnose preoperatively, and even transvaginal ultrasound in older women has detected only about 44% of paratubal cysts preoperatively [6]. Doppler study may show high impedance or absence of flow in a tubular structure. CT findings of IFTT include an adnexal cyst separate from the ovary, a twisted appearance to the fallopian tube, dilated tube greater than 15 mm, a thickened and enhancing tubal wall, and luminal CT attenuation greater than 50 H consistent with hemorrhage [9]. MRI abdomen is informative in complex situations when the ultrasound is indeterminate.

Laparoscopy is the gold standard in the diagnosis and management of paratubal cysts complicated with IFTT. Adnexal detorsion with resection of the paratubal cyst and preservation of the fallopian tube (if not gangrenous) is the procedure of choice. Fixation of the detorted tube is controversial. Surgery is usually done too late for tubal conservation. A few reports of detorsion salvaging the fallopian tube with subsequent pregnancy have been reported [10]. Salpingostomy, though unpopular for its potential to cause ectopic pregnancies, has been speculated to improve fertility, as a second stage management following salpingectomy [11].
